# Novel Regioselective Synthesis of Urolithin Glucuronides—Human Gut Microbiota Cometabolites of Ellagitannins and Ellagic Acid

**DOI:** 10.1021/acs.jafc.2c00170

**Published:** 2022-05-09

**Authors:** Jose M. Villalgordo, Laura Trulli, Rocío García-Villalba, Victor García, Yusuf Althobaiti, Francisco A. Tomás-Barberán

**Affiliations:** † Eurofins-VillaPharma Research S.L., Parque Tecnológico de Fuente Alamo, E-30320 Fuente Alamo, Murcia, Spain; ‡ Department of Pharmacology and Toxicology, College of Pharmacy, Taif University, P.O. Box 11099, Taif 21944, Saudi Arabia; § CEBAS-CSIC, Research Group on Quality, Safety, and Bioactivity of Plant-Derived Foods, P.O. Box 164, Espinardo, Murcia 30100, Spain

**Keywords:** ellagic acid, gut microbiota metabolites, urolithins, glucuronides, synthesis

## Abstract

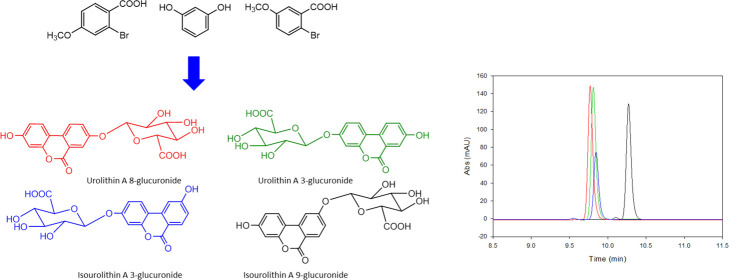

Urolithins (dibenzo-pyran-[*b*,*d*]-6 one derivatives) are human gut microbiota metabolites produced from the natural food antioxidant ellagic acid. Urolithins are better absorbed than ellagic acid and demonstrate biological activities that suggest that they are responsible for the health effects observed after consuming ellagitannin- and ellagic acid-containing foods. Urolithins occur in the systemic circulation as glucuronide conjugates following phase II metabolism. These phase II conjugates are essential for testing the urolithin mechanisms of action in human cell line bioassays. Urolithin glucuronides are not commercially available, and their biosynthesis leads to mixtures of regional isomers. This study describes a novel and regioselective synthesis of urolithin A (3,8-dihydroxy urolithin) 3- and 8-glucuronides and isourolithin A (3,9-dihydroxy urolithin) 3- and 9-glucuronides. The metabolites were characterized using ^1^H and ^13^C NMR spectroscopy and UV spectrophotometry. The presence of these metabolites in human subjects belonging to different urolithin metabotypes was also investigated.

## Introduction

Urolithins (hydroxy-6*H*-dibenzo[*b*,*d*]pyran-6-one derivatives) are gut microbiota metabolites produced from the natural polyphenolic antioxidants ellagic acid and ellagitannins.^1^ These antioxidants are present in significant quantities in foods, including pomegranates, muscadine grapes, berries (strawberries, raspberries, and blackberries), nuts (walnuts and pecans), tropical fruits (camu-camu and jaboticaba), tea, oak-aged wines, spirits, and many herbal medicinal products.^2,3^ Compared to ellagic acid, urolithins are better absorbed in humans and display biological activities that suggest that they are responsible for the health effects observed after consuming ellagitannin-containing foods.^3,4^ These include cardiovascular effects, anticancer activities, antiaging effects, and gut and systemic anti-inflammatory effects that also have impact in neurocognitive disorders, as shown in recent literature reviews.^5−7^ In the systemic circulation, urolithins occur mainly as glucuronide conjugates following phase II metabolism, enhancing their solubility and, therefore, their urinary excretion.^1,8,9^ Thus, these phase II conjugates are essential metabolites for testing the biological effects of urolithins on *in vitro* human cell line bioassays.^9,10^

The synthesis of glucuronide conjugates of phenolic compounds (4-hydroxycinnamic, urolithin B, hydroxytyrosol, resveratrol, citrus flavanones) has been attempted using different approaches based on the use of methyl-2,3,4-tri-*O*-acetyl-1-*O*-(trichloroacetimidoyl)-α-d-glucuronide, although in these studies, regioisomeric mixtures were obtained.^11−13^

**Figure 1:**
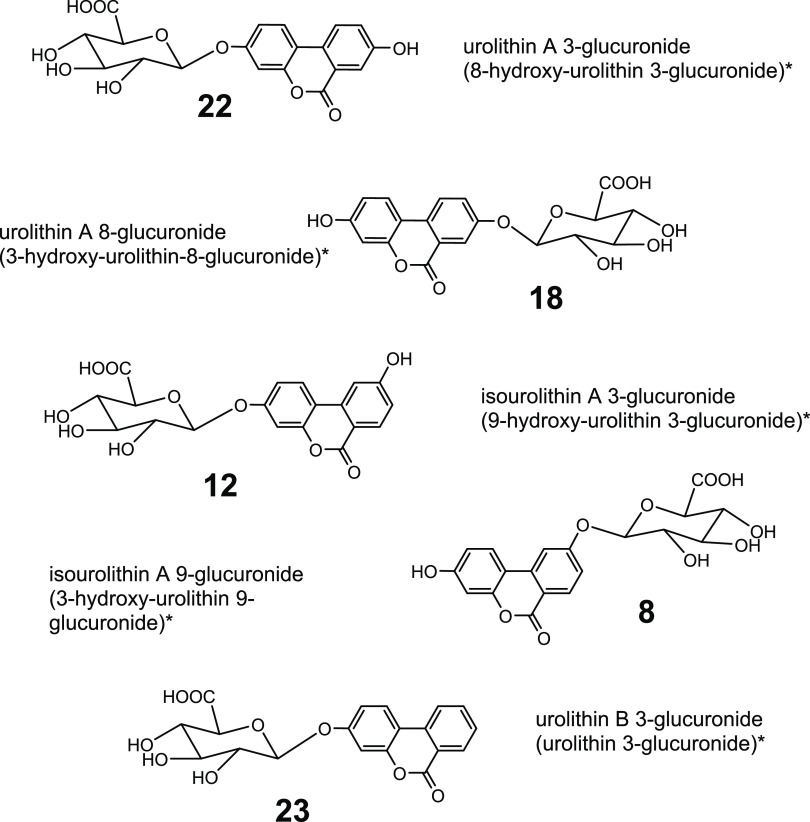
Structures of the urolithin glucuronide metabolites. *(Nomenclature following Kay et al., 2020).^23^

The different regioisomeric urolithin glucuronides have not been available through chemical synthesis so far. In an early attempt, the preparation of both urolithin A 8- and urolithin A 3-glucuronide (18 and 22, respectively) was described but not in a regioselective manner. In turn, these urolithins were obtained as a mixture of both regioisomers.^14^ Urolithin aglycones have been chemically synthesized using different methods.^15−19^ However, syntheses of the main circulating glucuronide metabolites, as single individual regioisomers, have not been reported. In fact, only the synthesis of urolithin B-glucuronide (urolithin 3-glucuronide) (23) was previously reported^13^ because urolithin B has only one hydroxyl group for glucuronidation, and this makes the synthesis straightforward. However, syntheses of the main circulating glucuronide metabolites have not been reported, probably because of difficulties in producing and isolating the two isomers of urolithin A and those of isourolithin A (Figure 1).

These different glucuronides may exert dissimilar biological effects or be produced in diverse quantities in different individuals due to enzyme polymorphisms as was shown for hesperetin glucuronide conjugates.^20^ However, this has not been demonstrated yet for urolithins due to the lack of authentic standards for these metabolites.

This study describes a novel and regioselective synthesis of urolithin A 3- and 8-glucuronides and isourolithin A 3- and 9-glucuronides and their characterization using ^1^H ^13^C NMR spectroscopy, mass spectrometry, and UV spectrophotometry.

## Materials and Methods

### Reagents and Chemicals

All solvents and reagents were purchased from commercial sources and were analytically pure and used as purchased. NaOH, CuSO_4_, 2-bromo-4-methoxybenzoic acid, 2-bromo-5-methoxybenzoic acid, resorcinol, triisopropylsilyl chloride (TIPS-Cl), imidazole, dimethylformamide (DMF), BBr_3_, OEt_2_, dichloromethane (DCM), K_2_CO_3_, KF, MeOH, H_2_O, pivaloyl chloride, pyridine, and BF_3_ were bought from Sigma-Aldrich (St. Louis). Methyl-(2,3,4-tri-*O*-acetyl-α-d-glucopyranosyl trichloroacetimidate) uronate. CAS Number: 92420-89-8. From Combi-Blocks (San Diego, CA).

#### General Synthetic Procedure for Compound **3** (3-Hydroxy-9-methoxy-6*H*-dibenzo[*b*,*d*]pyran-6-one)

The mixture of 2-bromo-4-methoxybenzoic acid **1** (2.0 g, 8.65 mmol), resorcinol **2** (2. g, 18.17 mmol), and NaOH (0.72 g, 18.17 mmol) in H_2_O (9.1 mL) was heated at 80 °C for 30 min. Then, aqueous 5% CuSO_4_ (3.6 mL) was added to the mixture and heated for additional 10 min. HCl (37% 1.0 mL) was added, and the resulting precipitate was filtered and washed with H_2_O (5.00 mL) and MeOH (3.0 mL). The filter cake was transferred to a flask, and MeOH (10 mL) was added. The suspension in MeOH was stirred at 50 °C for 10 min, newly filtered and washed with Et_2_O, and dried under a high vacuum to yield compound **3** as a colorless powder (1.50 g., 72%). ESI-MS (*m*/*z*): [M + H]^+^ calcd, 242.06; found, 242.90

#### General Synthetic Procedure for Compound **4** (9-Methoxy-3-((triisopropylsilyl)oxy)-6*H*-benzo[*c*]chromen-6-one)

To an ice–water-cooled solution of **3** (0.365 g; 1.507 mmol) in dry DMF (8.0 mL) were sequentially added triisopropyl silyl chloride (0.969 mg, 4.521 mmol) and imidazole (0.205 g, 3.014 mmol) and stirred until reaching room temperature (rt) overnight. Additional triisopropyl silyl chloride (0.969 mg, 4.521 mmol) and imidazole (0.205 g, 3.014 mmol) were added at rt, and the reaction mixture was stirred for further 3 h. The solvent was removed under reduced pressure, and the residue was partitioned between CH_2_Cl_2_ and H_2_O. The organic layer was evaporated under reduced pressure, and the residue was newly partitioned between EtOAc and H_2_O, and the layers were separated. The organic layer was dried over MgSO_4_, filtered, and concentrated under reduced pressure. The resulting crude material was purified by MPLC (SiO_2_, EtOAc/heptane 0:100 until 15:85) to afford **4** as a colorless solid (0.597 g, 94% yield). ESI-MS (*m*/*z*): [M + H]^+^ calcd, 398.19; found, 399.10.

#### General Synthetic Procedure for Compound **5** (9-Hydroxy-3-((triisopropylsilyl)oxy)-6*H*-benzo[*c*]chromen-6-one)

To a cooled (−80 °C) solution of **4** (1.300 g, 3.262 mmol) in dry CH_2_Cl_2_ (90.0 mL), BBr_3_ (1.886 mL, 19.572 mmol) was added dropwise. The reaction mixture was gradually warmed to rt and stirred for 12 h. The reaction mixture was poured into ice/water (50 mL) and extracted with EtOAc (3 × 30 mL). The combined organic layers were dried over Mg_2_SO_4_, filtered, and then concentrated under reduced pressure. The resulting crude material was purified by MPLC (SiO_2_, EtOAc/heptane 0:100 until 15:85) to afford **5** as a colorless solid (0.711 g, 70% yield). ESI-MS (*m*/*z*): [M + H]^+^ calcd, 384.55; found, 385.30.

#### General Synthetic Procedure for Compound **7** (2*S*,3*S*,4*S*,5R,6*R*)-2-(Methoxycarbonyl)-6-(((6-oxo-3-(triisopropylsilyl)oxy)-6*H*-benzo[*c*]chromen-9-yl)oxy)tetrahydro-2*H*-pyran-3,4,5-triyltriacetate

A freshly prepared 0.1 M solution in CH_2_Cl_2_ of BF_3_. Et_2_O (1.43 mL) was added to a CH_2_Cl_2_ (2 mL) solution of **6** (0.274 g, 0.572 mmol) and **5** (0.220 g, 0.572 mmol) in dry CH_2_Cl_2_ (2.0 mL) at rt. The mixture was stirred for 1 h at rt. The reaction mixture was concentrated under reduced pressure, and the resulting crude material was purified by MPLC (SiO_2_, EtOAc/heptane 0:100 until 30:70) to afford **7** as colorless foam (0.289 g, 72% yield). ESI-MS (*m*/*z*): [M + H]^+^ calcd, 700.82; found, 543.00 [M-TiPS]^+^.

#### General Synthetic Procedure for Compound **8** (2*S*,3*S*,4*S*,5*R*,6*R*)-3,4,5-Trihydroxy-6-((3-hydroxy-6-oxo-6*H*-benzo[*c*]chromen-9-yl)oxy)tetrahydro-2*H*-pyran-2-carboxylic Acid

To a mixture of **7** (0.289 g, 0.412 mmol), KF (0.048 g, 0.824 mmol), and K_2_CO_3_ (0.114 g, 0.824 mmol), MeOH–H_2_O (10 mL, 5:1) was added. The resulting mixture was stirred at rt for 16 h. The solvent was removed under vacuum, and the mixture was dissolved in H_2_O and purified by RP-HPLC to afford **8** (0.080 g, 48% yield). ESI-MS (*m*/*z*): [M + H]^+^ calcd, 404.33; found, 403.00 [M – H]^+^.

#### General Synthetic Procedure for Compound **9** (6-Oxo-3-((triisopropylsilyl)oxy)-6H-benzo[*c*]chromen-9-yl pivalate)

To a solution of **5** (0.146 g, 0.38 mmol) in pyridine (10 mL), pivaloyl chloride (0.935 mL, 7.60 mmol) was added dropwise at rt. The resulting mixture was stirred at rt for 18 h. The solvent was removed under reduced pressure, and the resulting crude material was purified by MPLC (SiO_2_, EtOAc/heptane 0:100 until 90:10) to afford **9** as a colorless solid (0.157 g, 88% yield). ESI-MS (*m*/*z*): [M + H]^+^ calcd, 468.23; found, 469.30.

#### General Synthetic Procedure for Compound **10** (3-Hydroxy-6-oxo-6*H*-benzo[*c*]chromen-9-yl pivalate)

To a solution of **9** (0.157 g, 0.335 mmol) in methanol (3.5 mL), KF (0.021 g, 0.368 mmol) was added. The resulting mixture was stirred at rt for 1 h. The solvent was removed under reduced pressure, and the resulting crude material was purified by MPLC (SiO_2_, EtOAc/heptane 0:100 until 20:80) to afford **9** as a colorless solid (0.062 g, 59% yield). ESI-MS (*m*/*z*): [M + H]^+^ calcd, 312.23; found, 311.00 [M – H]^+^.

#### General Synthetic Procedure for Compound **11** ((2*S*,3*S*,4*S*,5*R*,6*R*)-2-(Methoxycarbonyl)-6-((6-oxo-9-(pivaloyloxy)-6*H*-benzo[*c*]chromen-3-yl)oxy) tetrahydro-2*H*-pyran-3,4,5-triyltriacetate)

A freshly prepared 0.1 M solution in CH_2_Cl_2_ of BF_3_. Et_2_O (0.5 mL) was added to a CH_2_Cl_2_ (2 mL) solution of **6** (0.095 g, 0.199 mmol) and **10** (0.062 g, 0.199 mmol) in dry CH_2_Cl_2_ (2.0 mL) at room temperature. The mixture was stirred for 1 h at rt. The reaction mixture was concentrated under reduced pressure, and the resulting crude material was purified by MPLC (SiO_2_, EtOAc/heptane 0:100 until 30:70) to afford **7** as colorless foam (0.089 g, 72% yield). ESI-MS (*m*/*z*): [M + H]^+^ calcd, 628.18; found, 543.00 [M-*t*-BuCO]^+^.

#### General Synthetic Procedure for Compound **12** ((2*S*,3*S*,4*S*,5*R*,6*R*)-3,4,5-Trihydroxy-6-((9-hydroxy-6-oxo-6*H*-benzo[*c*]chromen-3yl)oxy) tetrahydro-2*H*-pyran-2-carboxylic Acid)

To a mixture of **11** (0.084 g, 0.134 mmol), KF (0.016 g, 0.268 mmol), and K_2_CO_3_ (0.037g, 0.268 mmol), MeOH–H_2_O (2,8 mL, 5:1) was added. The resulting mixture was stirred at rt for 16 h. The solvent was removed under vacuum, and the mixture was dissolved in H_2_O and purified by RP-HPLC to afford **12** (0.032 g., 59% yield). ESI-MS (*m*/*z*): [M + H]^+^ calcd, 404.33; found, 403.00 [M – H]^+^.

#### General Synthetic Procedure for Compound **14** (3-Hydroxy-8-methoxy-6*H*-dibenzo[*b*,*d*]pyran-6-one)

The mixture of 2-bromo-5-methoxybenzoic acid **13** (0.500 g., 2.164. mmol), resorcinol **2** (1.430. g, 12.98 mmol), and NaOH (0.672 g., 16.80 mmol) in H_2_O (25 mL) was refluxed for 1 h. Then, aqueous 28% CuSO_4_ (25 mL) was added to the mixture and heated for an additional 10 min. On cooling to rt, the precipitate formed was filtered and washed several times with ice–H_2_O and finally with Et_2_O and dried under high vacuum to yield compound **14** as a colorless powder (0.476 g., 91%). ESI-MS (*m*/*z*): [M + H]^+^ calcd, 242.06; found, 243.00

#### General Synthetic Procedure for Compound **15** (8-Methoxy-3-((triisopropylsilyl)oxy)-6H-benzo[*c*]chromen-6-one)

To an ice–water-cooled solution of 3-hydroxy-8-methoxy-6*H*-dibenzo[*b*,*d*]pyran-6-one, **3**, (0.050 g; 0.206 mmol) in dry DMF (8.0 mL) were sequentially added triisopropyl silyl chloride (0.132 mg, 0.618 mmol) and imidazole (0.028 g, 0.412 mmol) and stirred until rt overnight. The solvent was removed under reduced pressure, and the residue was partitioned between CH_2_Cl_2_ and H_2_O. The organic layer was evaporated under reduced pressure, and the residue was newly partitioned between EtOAc and H_2_O, and the layers were separated. The organic layer was dried over MgSO_4_, filtered, and concentrated under reduced pressure. The resulting crude material was purified by MPLC (SiO_2_, EtOAc/heptane 0:100 until 15:85) to afford **15** as a colorless solid (0.069 g, 84% yield). ESI-MS (*m*/*z*): [M + H]^+^ calcd, 398.19; found, 399.10.

#### General Synthetic Procedure for Compound **16** (8-Hydroxy-3-((triisopropylsilyl)oxy)-6H-benzo[*c*]chromen-6-one)

To a cooled (−80 °C) solution of 9-methoxy-3-((triisopropylsilyl)oxy)-6*H*-benzo[*c*]chromen-6-one **4** (1.0 g, 2.509 mmol) in dry CH_2_Cl_2_ (89 mL), BBr_3_ (1.45 mL, 15.05 mmol) was added dropwise. The reaction mixture was gradually warmed to rt and stirred for 12 h. The reaction mixture was poured into ice/water and extracted with CH_2_Cl_2_ (2×) and EtOAc (1×). The combined organic layers were dried over Mg_2_SO_4_, filtered, and then concentrated under reduced pressure. The resulting crude material was purified by MPLC (SiO_2_, EtOAc/heptane 0:100 until 10:90) to afford **16** as a colorless solid (1.0 g, 73% yield). ESI-MS (*m*/*z*): [M + H]^+^ calcd, 384.55; found, 385.40.

#### General Synthetic Procedure for Compound **17** ((2*S*,3*S*,4*S*,5*R*,6*R*)-2-(Methoxycarbonyl)-6-((6-oxo-3-((triisopropylsilyl)oxy)-6*H*-benzo[*c*]chromen-8-yl)oxy)tetrahydro-2*H*-pyran-3,4,5-triyl triacetate)

A freshly prepared 0.1 M solution in CH_2_Cl_2_ of BF_3_.Et_2_O (0.940 mL) was added to a CH_2_Cl_2_ (3 mL) solution of **6** (0.224 g, 0.468 mmol) and **16** (0.180 g, 0.468 mmol) in dry CH_2_Cl_2_ (2.0 mL) at room temperature. The mixture was stirred for 30 min at rt. The reaction mixture was concentrated under reduced pressure, and the resulting crude material was purified by MPLC (SiO_2_, EtOAc/heptane 0:100 until 30:70) to afford **17** as a colorless solid (0.248 g, 76% yield). ESI-MS (*m*/*z*): [M + H]^+^ calcd, 700.26; found, 542.90 [M-TiPS]^+^.

#### General Synthetic Procedure for Compound **18** ((2*S*,3*S*,4*S*,5*R*,6*R*)-3,4,5-Trihydroxy-6-((3-hydroxy-6-oxo-6*H*-benzo[*c*]chromen-8-yl)oxy) tetrahydro-2*H*-pyran-2-carboxylic Acid)

To a mixture of **17** (0.294 g, 0.419 mmol), KF (0.048 g, 0.838 mmol), and K_2_CO_3_ (0.115g, 0.838 mmol), MeOH–H_2_O (20 mL, 5:1) was added. The resulting mixture was stirred at rt for 16 h. The solvent was removed under vacuum, and the mixture was dissolved in H_2_O and purified by RP-HPLC to afford **18** as a colorless solid (0.078 g, 46% yield). ESI-MS (*m*/*z*): [M + H]^+^ calcd, 404.33; found, 403.00 [M – H]^+^.

#### General Synthetic Procedure for Compound **1**9 (3-Hydroxy-6-oxo-6*H*-benzo[*c*]chromen-8-yl pivalate)

To a solution of **16** (0.050 g, 0.107 mmol) in pyridine (3 mL), pivaloyl chloride (0.320 mL, 2.60 mmol) was added dropwise at rt. The resulting mixture was stirred at rt for 16 h. The solvent was removed under reduced pressure to afford a solid that was filtered and washed with cold MeOH to afford **19** as a colorless solid (0.043 g, 71% yield). ESI-MS (*m*/*z*): [M + H]^+^ calcd, 468.23; found, 469.20.

#### General Synthetic Procedure for Compound **20** (3-Hydroxy-6-oxo-6*H*-benzo[*c*]chromen-8-yl pivalate)

To a solution of **19** (0.050 g; 0.107 mmol) in MeOH (1 mL) was added KF (0.006 mg, 0.107 mmol). The reaction mixture was stirred at rt for 2 h. The solvent was evaporated under reduced pressure, and the resulting crude material was purified by MPLC (SiO_2_, EtOAc/heptane 0:100 until 50:50) to afford **20** as a colorless solid (0.025 g, 75% yield). ESI-MS (*m*/*z*): [M + H]^+^ calcd, 310.32, found, 313.00.

#### General Synthetic Procedure for Compound **21** ((2*S*,3*S*,4*S*,5*R*)-2-(Methoxycarbonyl)-6-((6-oxo-8-(pivaloyloxy)-6*H*-benzo[*c*]chromen-3-yl)oxy)tetrahydro-2*H*-pyran-3,4,5-triyl Triacetate)

A freshly prepared 0.1 M solution in CH_2_Cl_2_ of BF_3_·Et_2_O (2.1 mL) was added to a CH_2_Cl_2_ (2 mL) solution of **6** (0.398 g, 0.832 mmol) and **20** (0.260 g, 0.832 mmol) in dry CH_2_Cl_2_ (3.0 mL) at 0 °C. The mixture was stirred for 1 h at rt. The reaction mixture was concentrated under reduced pressure, and the resulting crude material was purified by MPLC (SiO_2_, EtOA/heptane 0:100 until 50:50) to afford **21** as a colorless solid (0.179 g, 99% yield). ESI-MS (*m*/*z*): [M + H]^+^ calcd, 628.18; found, 646.40 [M + H_2_O]^+^.

#### General Synthetic Procedure for Compound **22** ((2*S*,3*S*,4*S*,5*R*,6*R*)-3,4,5-Trihydroxy-6-((8-hydroxy-6-oxo-6*H*-benzo[*c*]chromen-3-yl)oxy) tetrahydro-2*H*-pyran-2-carboxylic Acid)

To a mixture of **21** (0.318 g, 0.506 mmol), KF (0.059 g, 1.012 mmol), and K_2_CO_3_ (0.140 g, 1.012 mmol), MeOH–H_2_O (10 mL, 5:1) was added. The resulting mixture was stirred at rt for 16 h. The solvent was removed under vacuum, and the mixture was dissolved in H_2_O and purified by RP-HPLC to afford **12** (0.094 g., 46% yield). ESI-MS (*m*/*z*): [M + H]^+^ calcd, 404.07; found, 403,10 [M – H]^+^.

### HPLC–DAD-MS Analysis

Analysis of the synthesized standards and urine samples was developed using a 1200 HPLC chromatograph coupled in series with a photodiode array detector and a 6120 single-quadrupole mass spectrometer [HPLC–DAD-ESI-Q (MS)] (Agilent Technologies, Santa Clara CA). A method previously optimized for analyzing urolithins in biological samples was applied.^9^

### NMR Analyses

The NMR spectra were recorded on a Brüker 500 MHZ Advance with a Cryofit (Bruker, Bremen, Germany) in dimethyl sulfoxide-*d*_6_ (DMSO-*d*_6_) with tetramethylsilane (TMS) as an internal standard. ^1^H NMR, ^13^C NMR, and 2D-HSQC experiments were completed.

### Urine Collection from Volunteers Belonging to Urolithin Metabotypes A and B

Two healthy volunteers characterized as belonging to urolithin metabotypes A and B^19^ consumed 30 g of walnuts/day for 3 days. Walnuts were purchased at a local supermarket. After the last intake, a urine sample was collected and immediately stored at −20 °C, until analysis.^21^ Institutional ethical approvals were unnecessary as the experiments were carried out with freely available foodstuff, and only urine samples were collected, as advised by the Ethical Committee for previous studies.^22^ The volunteers gave written informed consent.

## Results and Discussion

### Synthesis of Isourolithin A (3,9-Dihydroxy Urolithin) Glucuronides

For isourolithin A 9-glucuronide (**8**), the synthetic sequence was initiated through the preparation of 9-*O*-methyl isourolithin A (**3**), in 72% yield, according to a known methodology based on the condensation between resorcinol (**2**) and a benzoic acid derivative (**1)**.^13,15,16^ Next, protection of the phenolic group using TiPS-Cl in the presence of imidazole afforded the corresponding derivative **4** in 94% isolated yield after chromatographic purification. Very important in this approach is that the triisopropylsilyl protecting group in **4** showed to be stable enough toward the demethylation reaction with BBr_3_ in DCM at 0 °C, thus leading to pure phenol derivative **5** in 70% isolated yield after chromatographic purification.

**Scheme 1:**
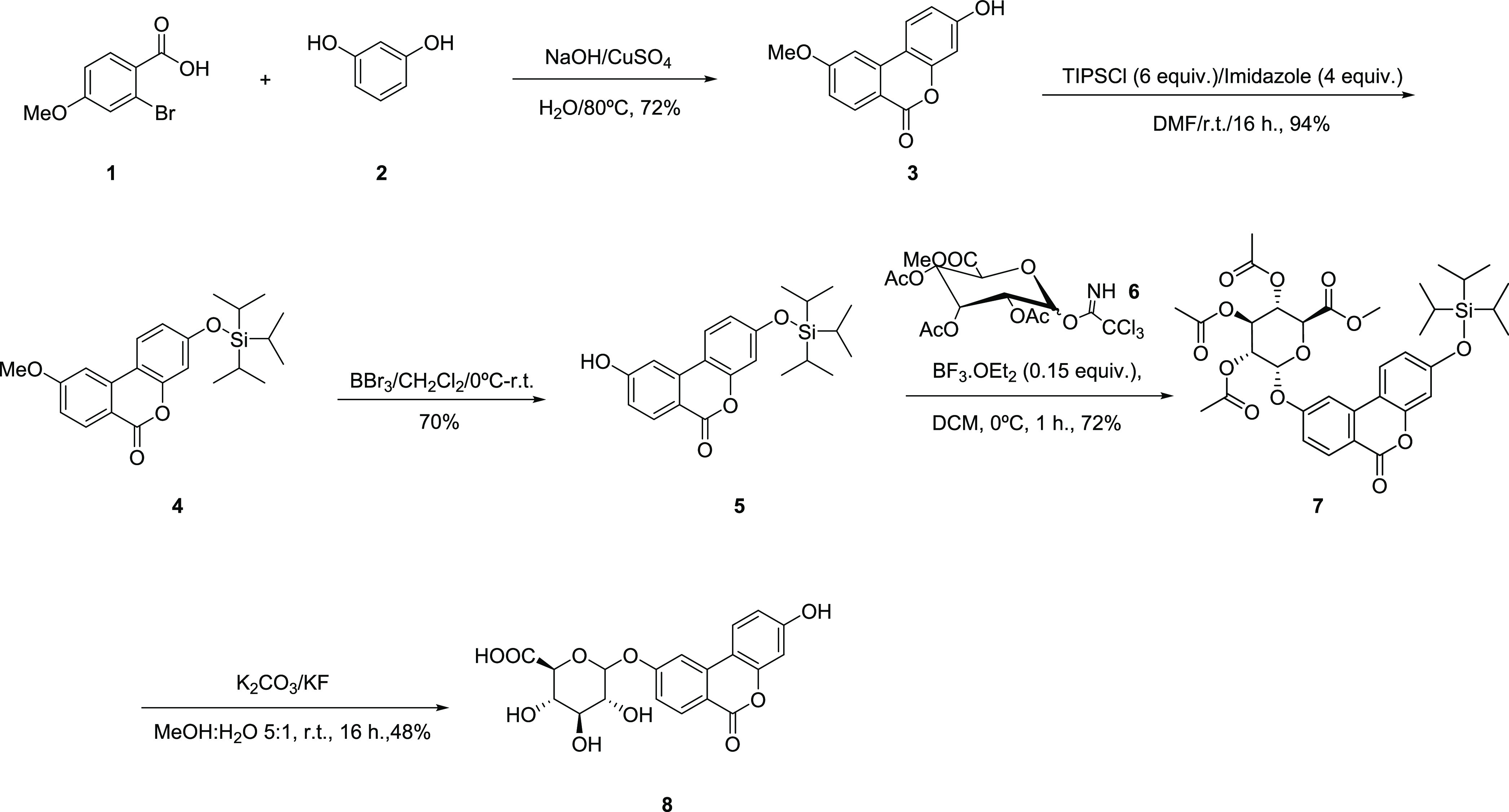
Synthesis of Isourolithin A 9-Glucuronide (**8**)

Finally, glycosylation of the acceptor isourolithin derivative **5** with commercially available glucuronosyl donor **6** in using BF_3_·OEt_2_ as a promoter^13^ afforded **7** in 72% isolated yield. One-pot, simultaneous desilylation/saponification reaction of **7** with KF/K_2_CO_3_ in MeOH–H_2_O afforded the desired isourolithin A 9-glucuronide (**8**) in 48% after RP-HPLC purification and with >99% purity (Scheme 1).

**Scheme 2:**
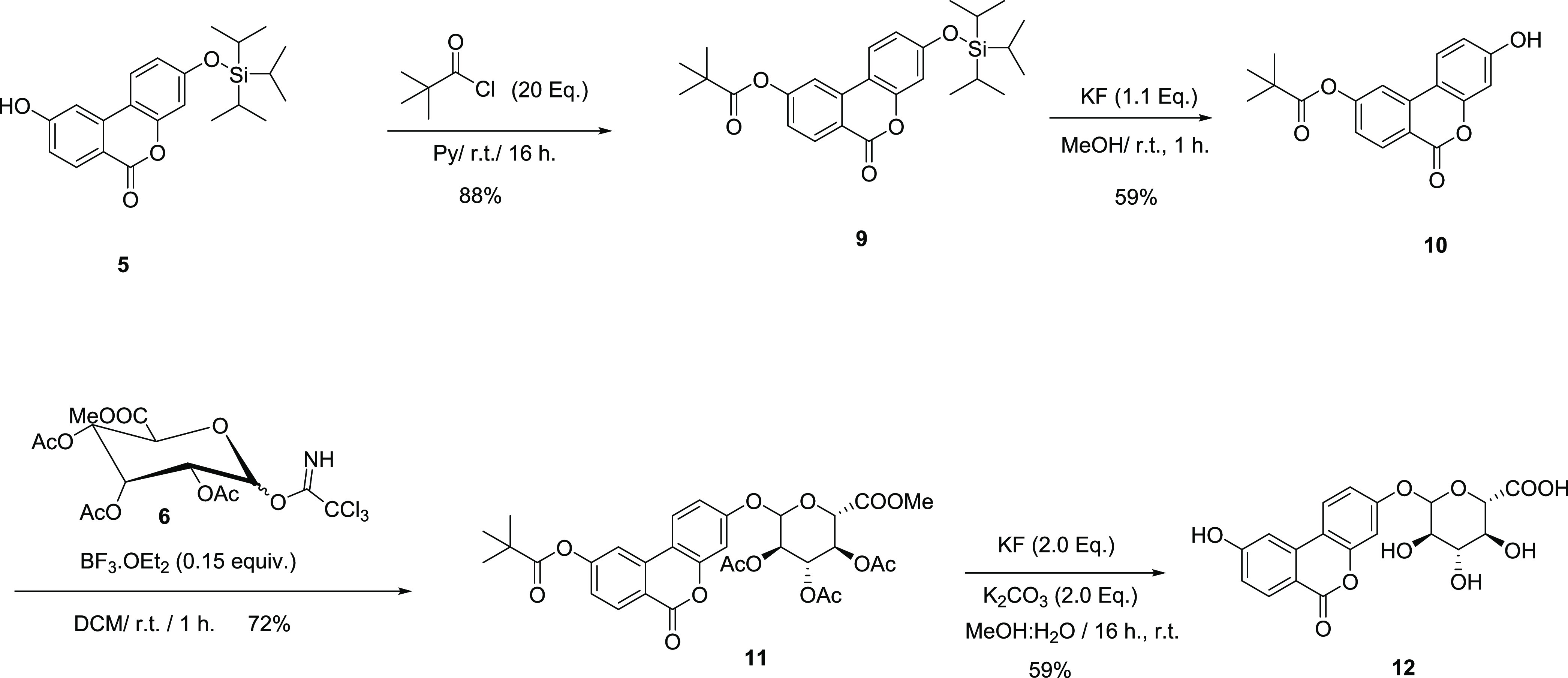
Synthesis of Isourolithin A 3-Glucuronide (**12**)

Alternatively, also from intermediate **5**, protection of the free 9-OH with pivaloyl chloride under standard conditions afforded the fully protected intermediate **9** in 88% yield. Selective deprotection of the silyl group with KF in MeOH afforded the desired regioisomeric phenol **10** in 59% yield with a free −OH group now at the 3-position. Glycosidation of **10** promoted by BF_3_·Et_2_O under analogous conditions as for isourolithin A 9-glucuronide **8** afforded the fully protected glucuronide **11** (72%), which was completely saponified in a one-pot reaction with KF/K_2_CO_3_ in MeOH/H_2_O to afford the desired isourolithin A 3-glucuronide (**12**) in 49% isolated yield after RP-HPLC purification and with >99% purity (Scheme 2).

#### Synthesis of Urolithin A (3,8-Dihydroxy Urolithin) Glucuronides

With a robust methodology in hands toward the regioselective preparation of isourolithin A 9- and 3-glucuronides in the pure form and satisfactory yields, **8** and **12**, respectively, we next addressed the synthesis of urolithin A 3- and 8-glucuronides using essentially an analogous approach.

**Scheme 3:**
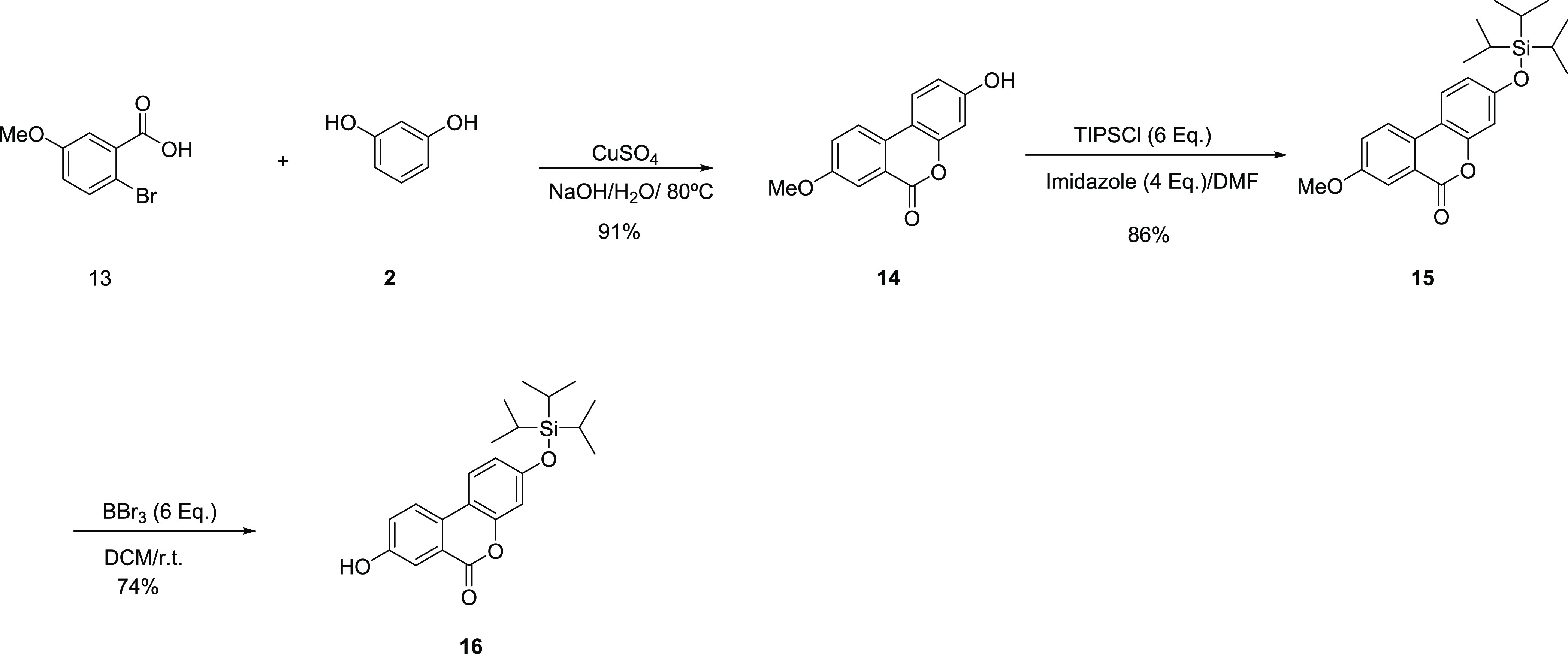
Synthesis of Urolithin A Intermediate **16**

Thus, from commercially available bromobenzoic acid (**13)** and resorcinol (**2**), 8-methoxy urolithin A (**14**) was obtained under standard conditions of CuSO_4_ in basic media, in 91% isolated yield.^13,15,16^ Protection with TiPS-chloride led to **15** that underwent selective demethylation with BBr_3_ in DCM, producing the desired key intermediate **16** (Scheme 3).

**Scheme 4:**
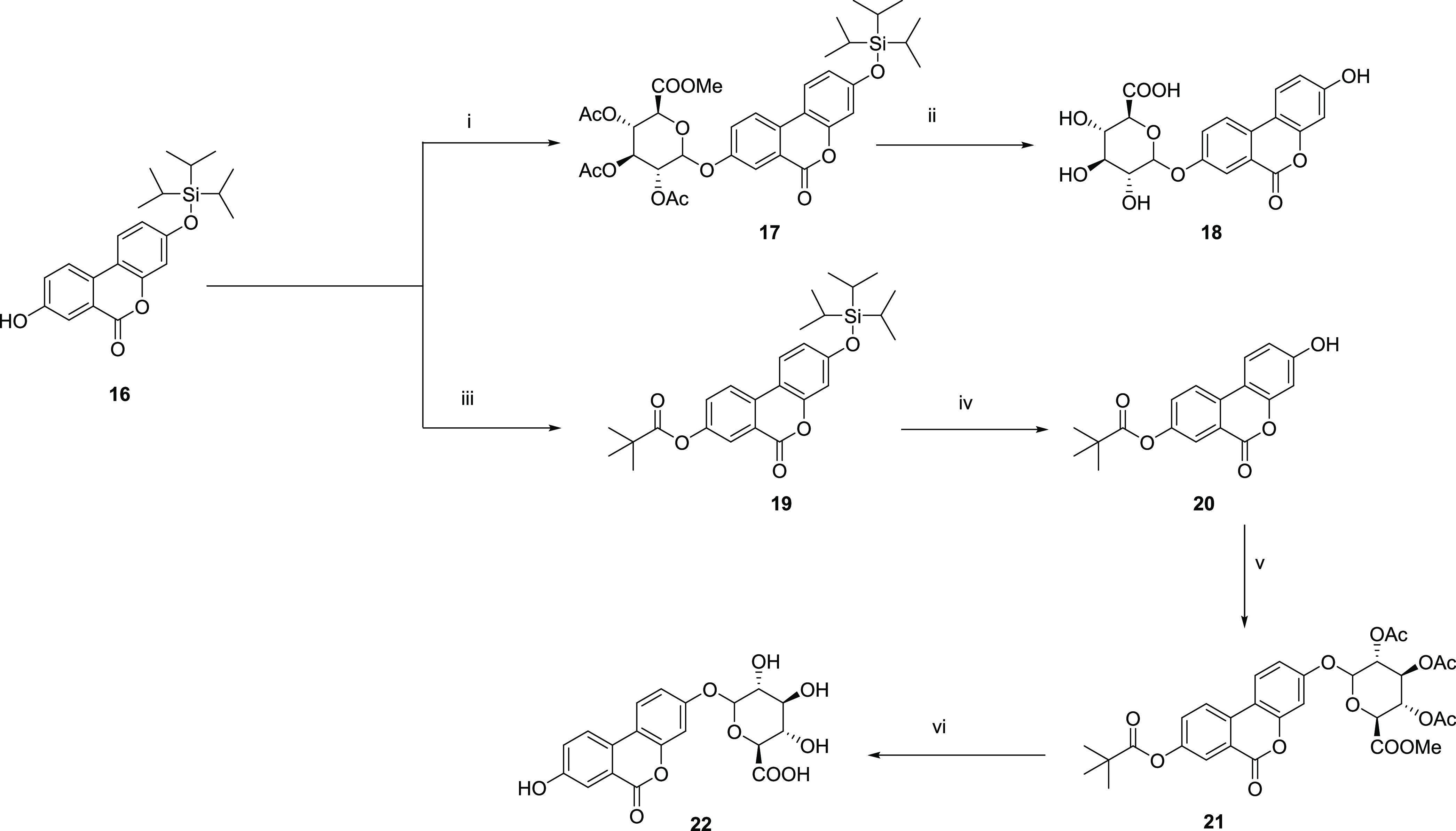
Synthesis of Urolithin A 8-Glucuronide (**18**) and 3-Glucuronide (**22**) Reagents and conditions: (i) 6, BF_3_.Et_2_O (0.15 equiv), CH_2_Cl_2_, rt, 1 h., 76% yield; (ii) KF (2 equiv), K_2_CO_3_ (2 equiv), MeOH/H_2_O, rt, 16 h., 46% yield; (iii) pivaloyl chloride (20 equiv), Py, rt, 16 h; 71% yield; (iv) KF (1.1 equiv), MeOH, rt, 1 h., 75% yield; (v) 6, BF_3_.Et_2_O (0.15 equiv), CH_2_Cl_2_, rt, 1 h, 99% yield; and (vi) KF (2 equiv), K_2_CO_3_ (2 equiv), MeOH/H_2_O (5:1), rt, 16 h, 46%.

Next, following the same strategy and analogous reaction conditions as those employed for isourolithin-A derivatives **8** and **12**, glycosylation of **16** under standard conditions afforded intermediate **17** in 76% yield, which after subsequent one-pot, full deprotection, led to urolithin A 8-glucuronide (**18**) as a single regioisomer in 46% yield, after RP-HPLC purification and with >99% purity (Scheme 4).

Alternatively, in turn, introduction of the pivaloyl group in **16** afforded **19** in 71% yield. Selective removal of the silyl protecting group using KF in MeOH led to the phenol **20** in 75% yield, which was subjected to glycosylation under analogous conditions to afford **21** and hence to the desired urolithin A 3-glucuronide (**22)** upon one-pot removal of the corresponding protecting groups in satisfactory overall yields and with >99% purity (Scheme 4).

### Characterization of the Synthesized Urolithin Glucuronide Conjugates

The structures of the synthesized metabolites (Figure 1) were confirmed by ^1^H NMR and ^13^C NMR (500 MHz). The NMR results of the glucuronides dissolved in DMSO-*d*_6_ are summarized in Tables 1 and 2. The chemical shifts were consistent with those previously published for the available urolithin glucuronides isolated from human urine.^24^ However, in this previous study, some of the metabolites were not isolated due to difficulties in the chromatographic separation of urolithin A 3-glucuronide (**22**), urolithin A 8-glucuronide (**18**), and isourolithin A 9-glucuronide (**12**), which coeluted as a single peak, and the ^1^H NMR of the mixture was reported.^24^ All the synthesized urolithin conjugates were β-glucuronides as the H-1 anomeric signal (GlucU 1 in Table 1) appeared as a doublet between 5.13 and 5.51 ppm. Coupling constants of about 7 Hz were consistent with those values previously reported for urolithin glucuronides.^24^ The downfield shifts for H-2 and H-4 signals of the 3-glucuronides (at 6.82–7.03 and 6.74–7.07 ppm, respectively) confirmed the position of glucuronidation, which was consistent with previous results.^24,25^ A similar behavior was observed for H-7 and H-9 of the 8-glucuronide (shifts at 7.54–7.72 and 7.35–7.55 ppm, respectively) and also for the H-8 and H-10 of the 9-glucuronide (shifts at 6.48–7.18 ppm and 6.65–7.76 ppm, respectively) when compared with the results for the corresponding metabolites with free hydroxyls at the 3-, 8-, or 9-positions (Table 1).

**Table 1:**
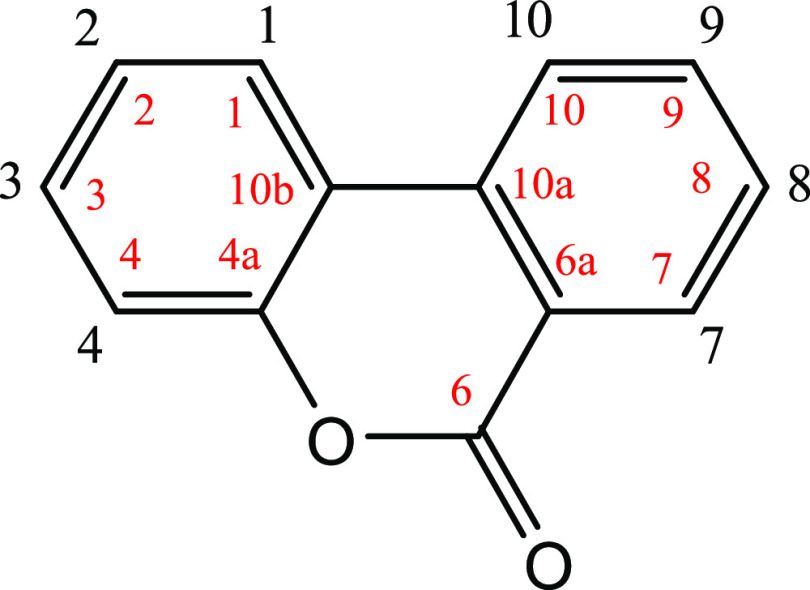
^1^H NMR Spectroscopy Data (500 MHz) DMSO-*d*_6_^a^

**metabolite**	**urolithin A 3-glucuronide**	**urolithin A 8-glucuronide**	**isourolithin A 3-glucuronide**	**isourolithin A 9-glucuronide**	**urolithin B 3-glucuronide**
	**8**	**12**	**18**	**22**	**23**
protons					
1	8.15 (d, *J* = 8.7)	8.09 (d, *J* = 8.8)	7.99 (d, *J* = 8.7)	8.16 (d, *J* = 8.8)	8.31 (d, *J* = 8.9)
2	7.03 (dd, *J* = 8.7, 2.4)	6.82 (dd, *J* = ′8.8, 2.3)	6.98 (dd, *J* = 8.7, 2.1)	6.83 (dd, *J* = 8.8, 2.3)	7.08 (dd, *J* = 8.9, 2.4)
3		10.27			
4	7.07 (d, *J* = 2.4)	6.74 (d, *J* = 2.3)	6.96 (d, *J* = 2.1)	6.72 (d, *J* = 2.3)	7.12 (d, *J* = 2.4)
7	7.54 (d, *J* = 2.7)	7.72 (d, *J* = 2.7)	7.97 (d, *J* = 8.8)	8.12 (d, *J* = 8.8)	8.36 (d, *J* = 8.3)
8			6.98 (dd, *J* = 8.8, 2.2)	7.18 (dd, *J* = 8.8, 2.2)	7.63 (t, *J* = 7.7)
9	7.35 (dd, *J* = 8.8, 2.7)	7.55 (dd, *J* = 8.9, 2.7)			7.93 (t, *J* = 7.7)
10	8.19 (d, *J* = 8.8)	8.22 (d, *J* = 8.9)	7.37 (d, *J* = 2.0)	7.76 (d, *J* = 2.2)	8.23 (d, *J* = 7.7)
GlucU 1	5.17 (d, *J* = 7.1)	5.17 (d, *J* = 7.1)	5.13 (d, *J* = 7.06)	5.42 (d, *J* = 7.0)	5.51 (d, *J* = 4.7)
GlucU 2	3.23–3.45	3.23–3.45	3.56–3.72	3.24–3.49	5.19–5.31
GlucU 3	3.23–3.45	3.23–3.45	3.56–3.72	3.24–3.49	3.34–3.40
GlucU 4	3.23–3.45	3.23–3.45	3.56–3.72	3.24–3.49	3.34–3.40
GlucU 5	3.94 (d, *J* = 9.1)	3.91 (d, *J* = 7.8)	3.95 (d, *J* = 9.4)	3.95 (d, *J* = 8.9)	4.01 (d, *J* = 9.3)

a d: Doublet; dd: double doublet.

**Table 2:**
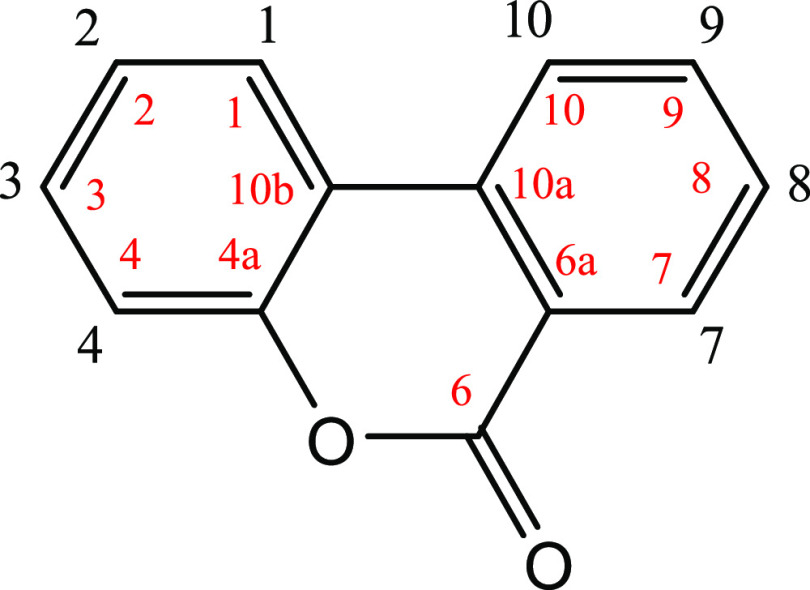
^13^C NMR Spectroscopy Data (500 MHz) DMSO*-d*_6_

**metabolite**	**urolithin A 3-glucuronide**	**urolithin A 8-glucuronide**	**isourolithin A 3-glucuronide**	**isourolithin A 9-glucuronide**	**urolithin B 3-glucuronide**
	8-hydroxy-urolithin 3-glucuronide	3-hydroxy-urolithin 8-glucuronide	9-hydroxy-urolithin 3-glucuronide	3-hydroxy urolithin 9-glucuronide	Urolithin 3-glucuronide^a^
carbons^b^					
1	124.19	124.82	124.79	125.61	125.29
2	114.09	113.62	113.85	113.49	114.04
3	157.94	159.69	159.24	162.83	159.06
4	104.33	103.34	104.15	103.33	104.34
4a	151.00	151.83	152.47	152.93	152.29
6	160.87	160.82	160.61	160.65	160.91
6a	121.21	120.53	111.04	113.46	119.99
7	113.98	115.38	132.67	132.59	130.19
8	158.03	156.67	117.52	117.18	128.89
9	124.59	125.34	164.63	160.58	135.86
10	124.55	124.09	106.98	107.36	122.63
10a	112.86	109.81	112.13	109.77	112.45
10b	126.72	130.18	137.15	137.74	135.00
GlucU 1	100.09	100.72	99.99	99.40	99.90
GlucU 2	73.35	73.45	73.41	73.37	73.31
GlucU 3	76.31	76.11	76.70	76.49	76.20
GlucU 4	71.86	71,87	72.29	71.92	71.76
GlucU 5	75.58	75.73	74.66	75.40	75.78
GlucU 6	170.82	170.75	172.22	171.00	170.59

a Nomenclature as Kay et al., 2020.^23^

b Carbon numbering (in red) as in Piwowarski et al.^24^

The ^13^C NMR analysis (Table 2), DEP, and the HSQC analyses (Supporting Information Figure 1) also confirmed the structure of the synthesized urolithin glucuronide conjugates.

**Figure 2:**
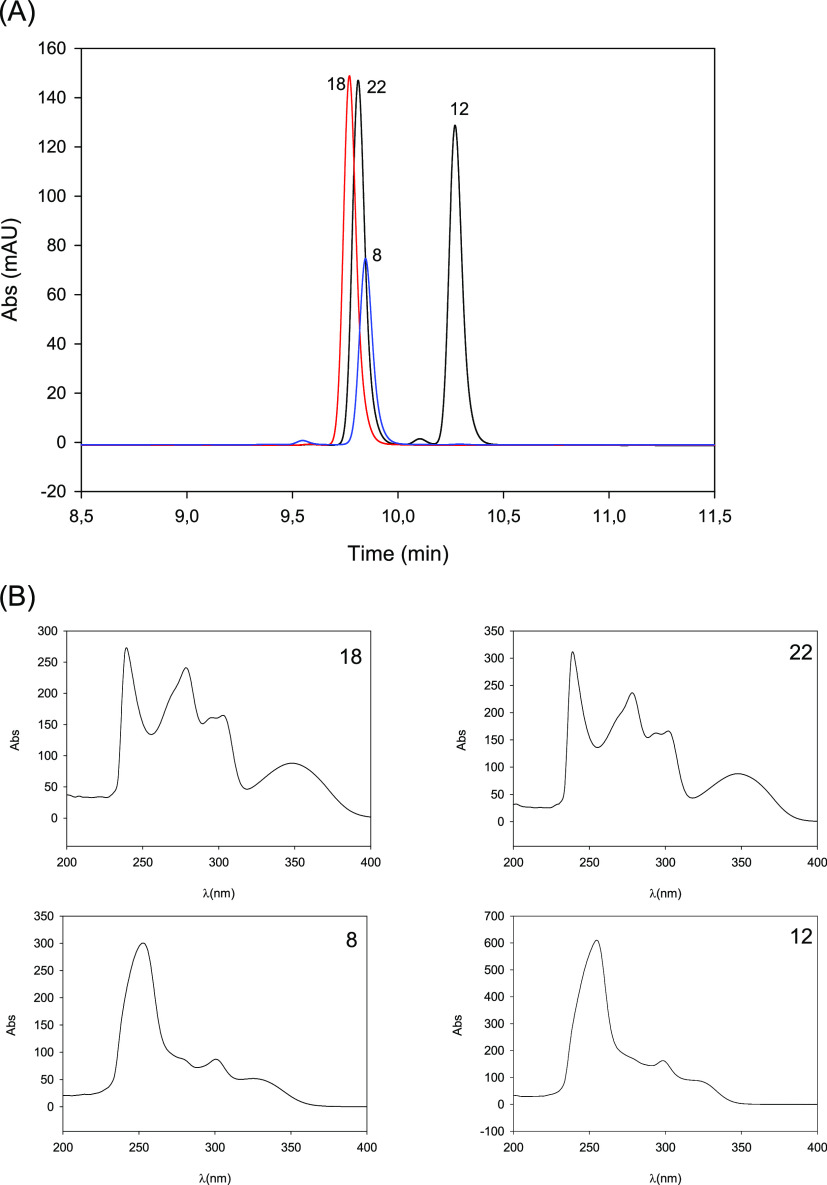
HPLC–DAD chromatogram (305 nm) (A) and UV spectra (B) of the synthesized urolithin glucuronides. Urolithin A 8-glucuronide (**18**); urolithin A 3-glucuronide (**22**); isourolithin A 9-glucuronide (**8**); and isourolithin A 3-glucuronide (**12**).

The HPLC analysis of the synthesized urolithin glucuronides also demonstrated that urolithin A 3-glucuronide (**22**) (8-hydroxy-urolithin 3-glucuronide) and the isomeric urolithin A 8-glucuronide (**18**) (3-hydroxy-urolithin-8-glucuronide) could not be resolved on reversed-phase columns. However, urolithin A 8-glucuronide eluted slightly earlier than the 3-glucuronide (Figure 2A). Isourolithin A 9-glucuronide (**8**) (3-hydroxy-urolithin 9-glucuronide) also eluted at a similar retention time, complicating the analysis. Only isourolithin A 3-glucuronide (**12**) (9-hydroxy-urolithin 3-glucuronide) and urolithin B glucuronide (**23**) (urolithin 3-glucuronide) were sufficiently resolved.

The UV spectra of the different metabolites recorded by HPLC–DAD were similar to those previously published,^9^ with the most remarkable differences observed between the spectra of the urolithin A (3,8-dihydroxy-urolithin) and isourolithin A (3,9-dihydroxy-urolithin) conjugates (Figure 2B). The MSMS spectra revealed similar fragmentation patterns for all the compounds with the main fragments at *m*/*z* 227 and 113, as previously reported.^9^

**Figure 3:**
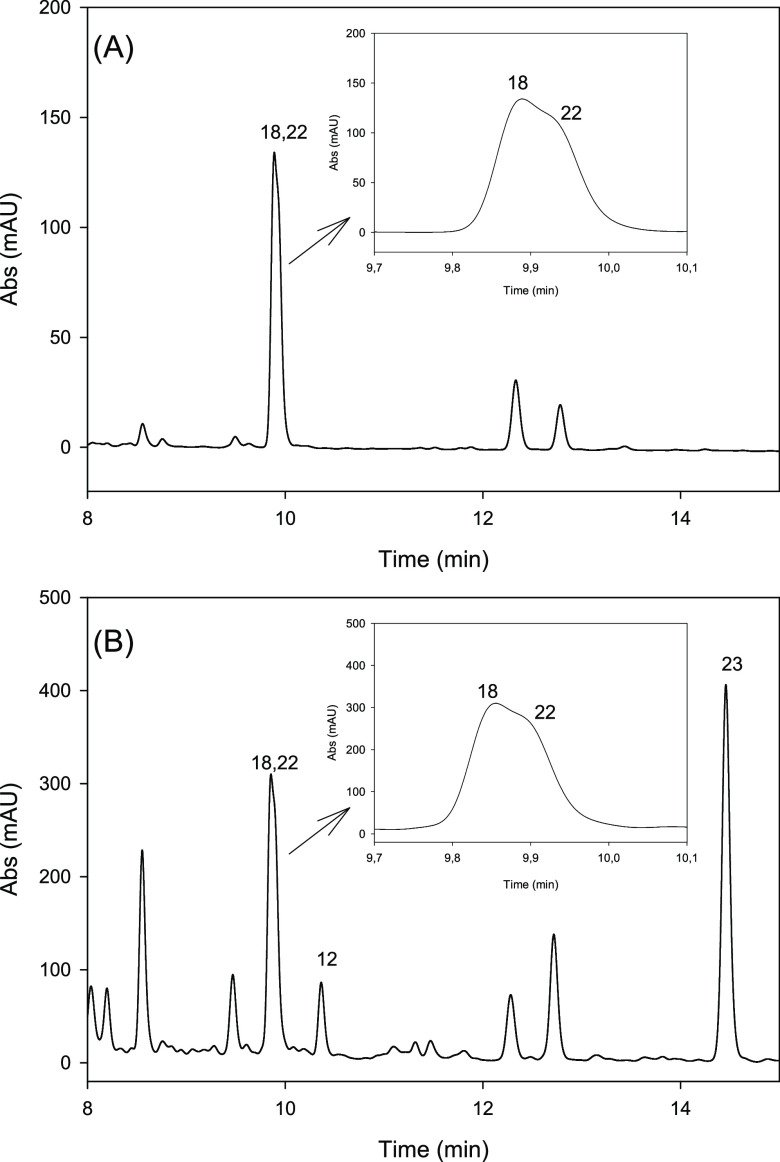
HPLC–DAD chromatograms at 305 nm of urine samples from volunteers belonging to urolithin metabotype A (A) and urolithin metabotype B (B). Urolithin A 8-glucuronide (**18**); urolithin A 3-glucuronide (**22**); isourolithin A 3-glucuronide (**12**); and urolithin B 3-glucuronide (**23**).

Urine samples from individuals belonging to the main urolithin metabotypes A and B^21^ were collected after walnut ellagitannin intake (30 g walnuts for 3 days) and analyzed by HPLC (Figure 3). The results showed that isourolithin A 3- (**12**) and 9 -glucuronides (**8**) separated neatly. However, isourolithin A 9-glucuronide was not visible in the chromatogram from metabotype B urine as coeluted with the urolithin A glucuronides (**18** and **22**) (Figure 3B). The analysis of urine from the metabotype A individual revealed that the two urolithin A conjugates coeluted in a broad peak. Both metabolites were visible although not resolved in the chromatographic peak (Figure 3A). Urolithin A 8-glucuronide eluted first (**18**), and 3-glucuronide (**22**) eluted as a shoulder of the 8-glucuronide chromatographic peak.

These results indicate that further studies are required to optimize the separation of all the possible urolithin conjugates in biological samples, to improve the urolithin metabotype assignment of individuals and to explore the glucuronyl transferase polymorphisms^20^ that can also affect inter-individual variations in ellagitannin metabolism and their effects in human health.^26,27^

## Supporting Information Available

Full 2D-HSQC experiments of urolithin B 3-glucuronide and urolithin A 8-glucuronide (PDF)
