# Supplementary figures and images for: Novel Regioselective Synthesis of Urolithin Glucuronides—Human Gut Microbiota Cometabolites of Ellagitannins and Ellagic Acid
Source: J Agric Food Chem. 2022 May 9;70(19):5819–28. doi: 10.1021/acs.jafc.2c00170 (PMC9121390; doi:10.1021/acs.jafc.2c00170)

Supplementary Figure 1

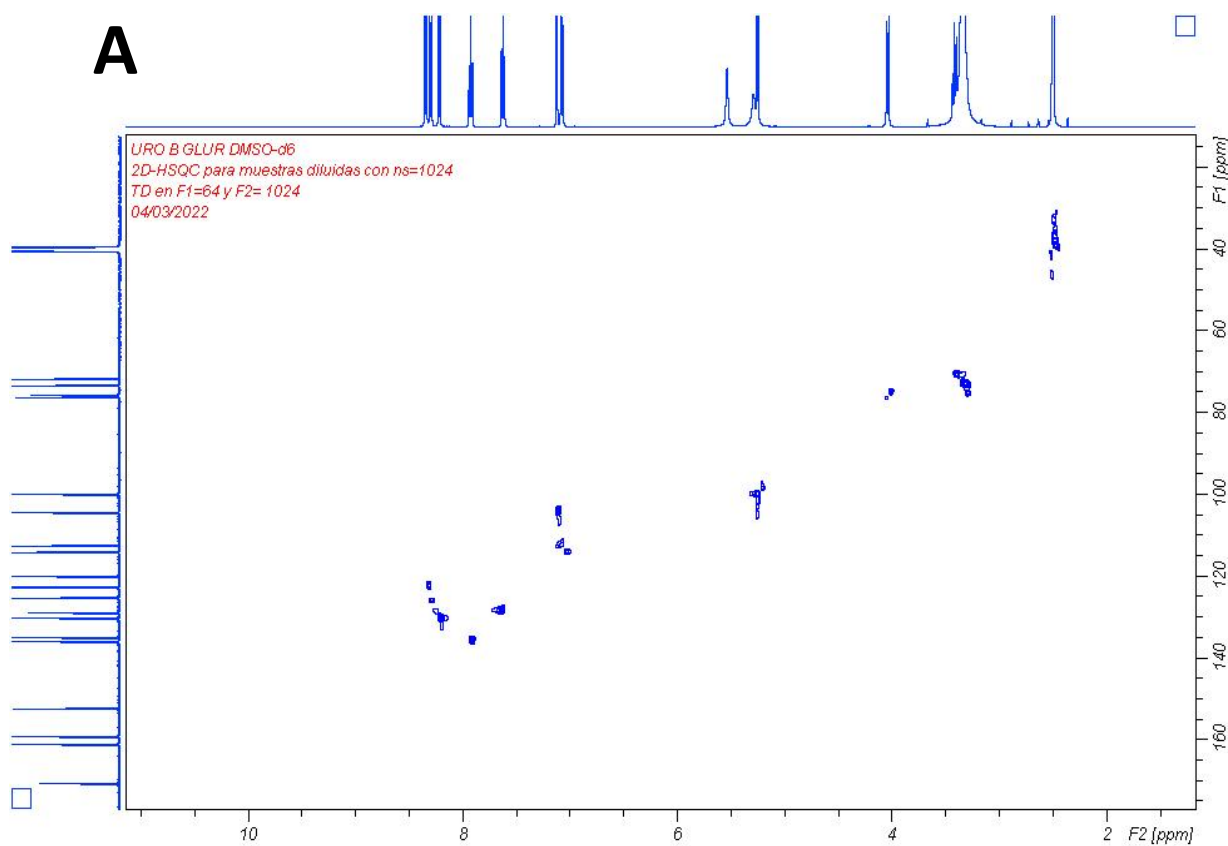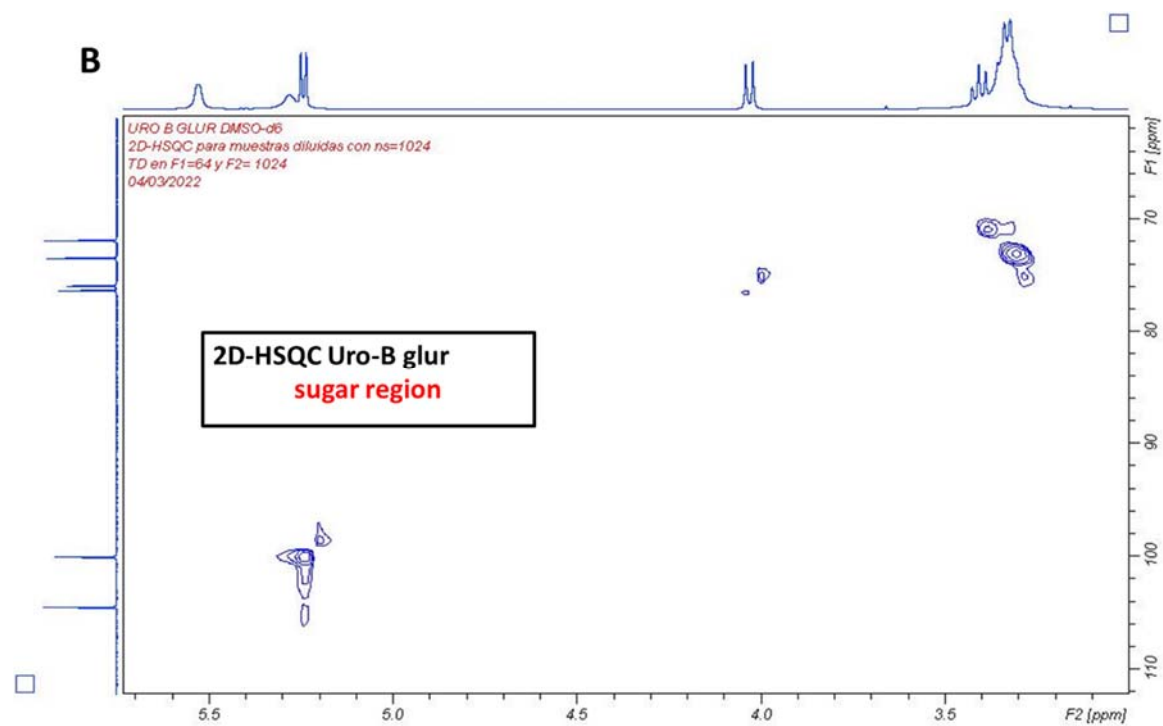

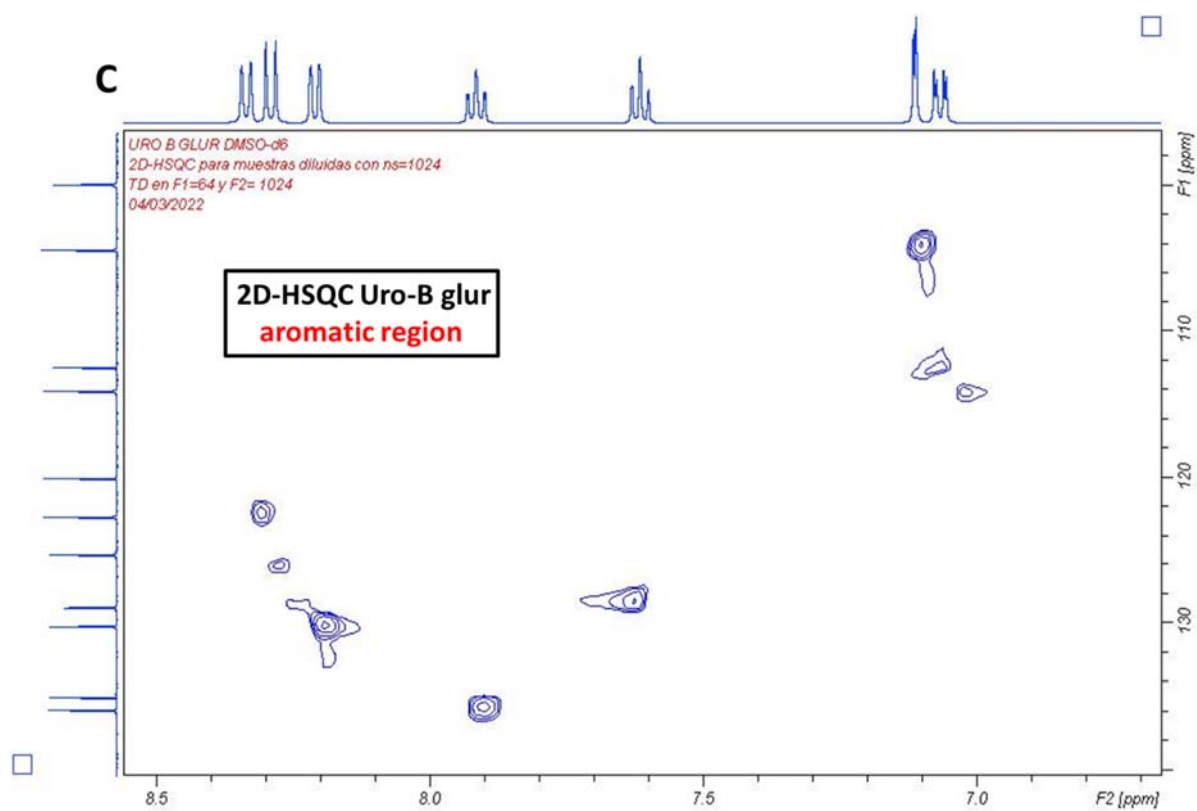

Supplementary Figure 2.

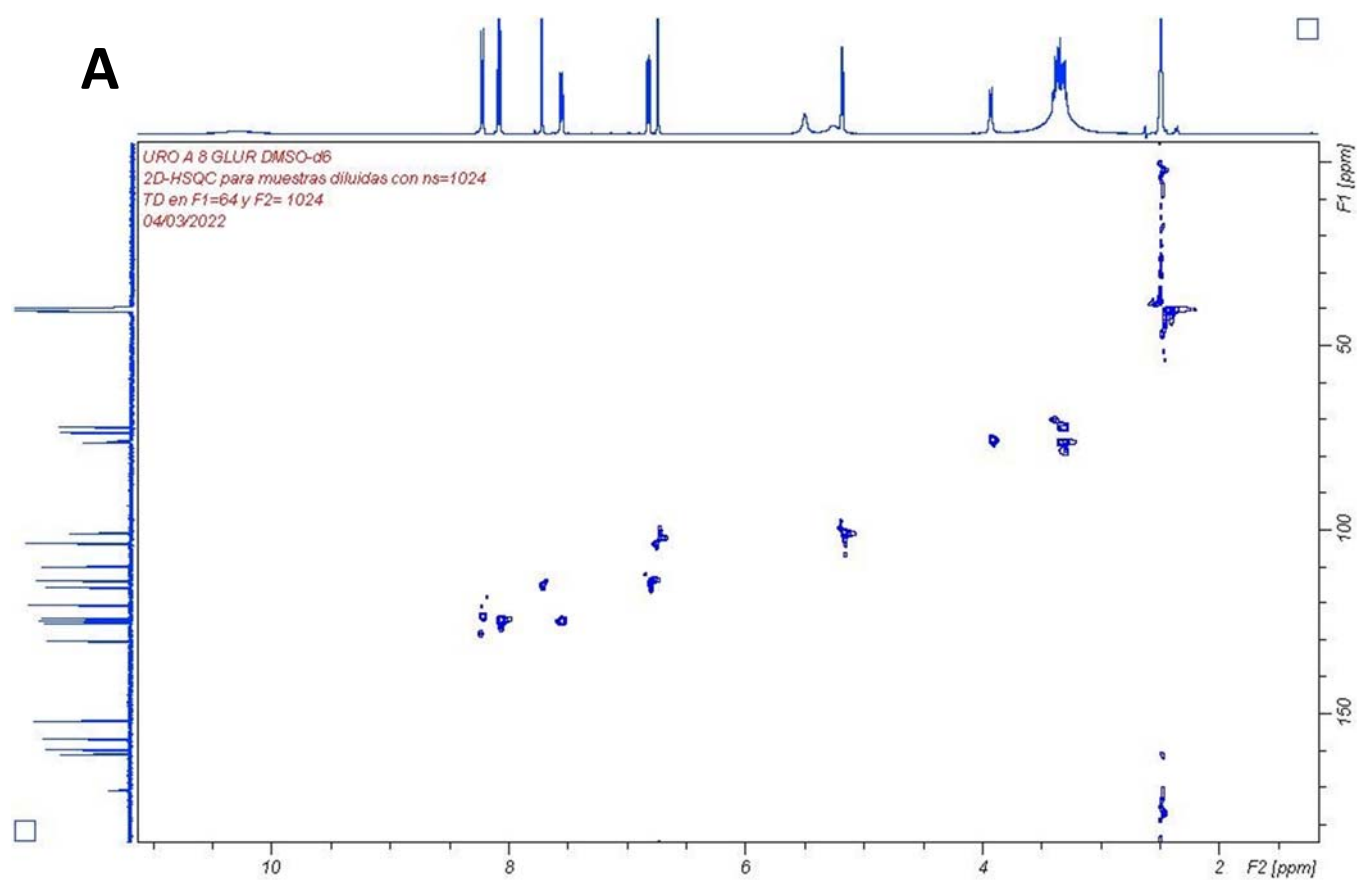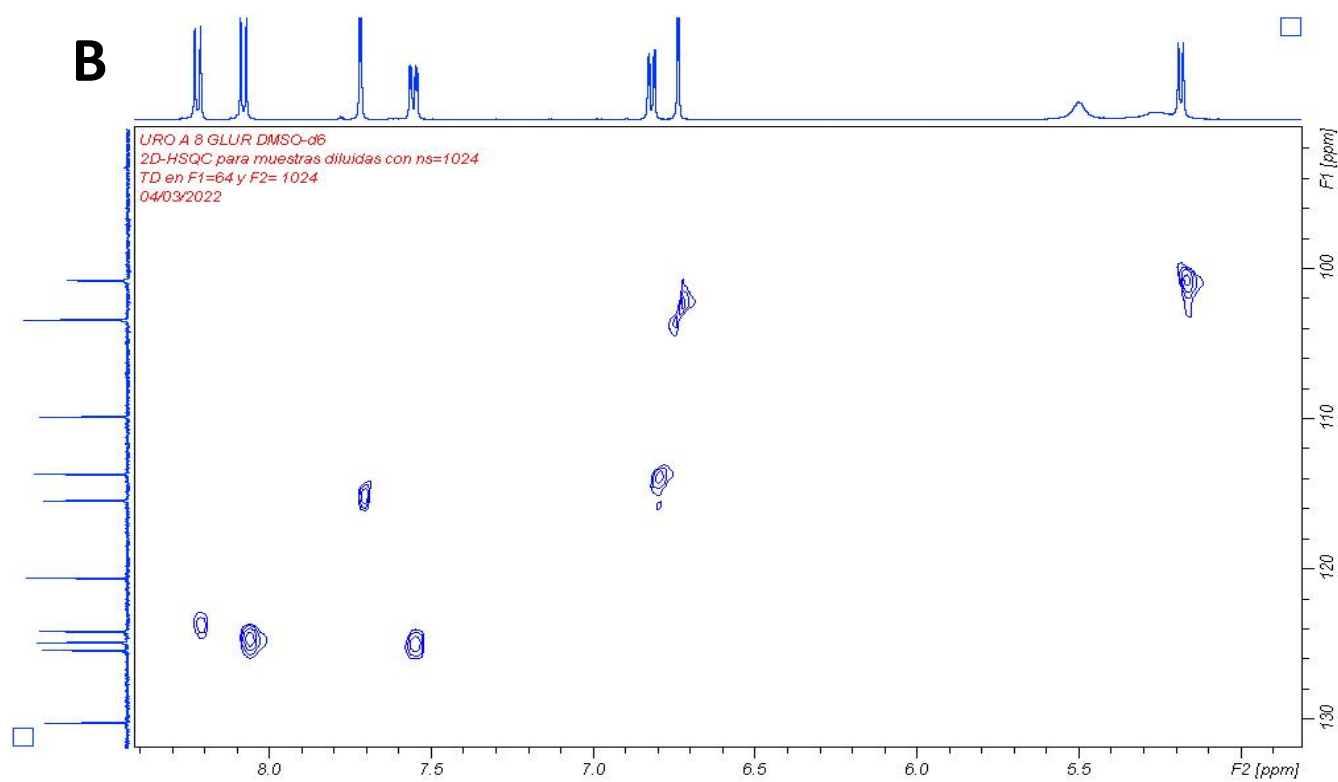

Supplement: Supplementary file 1 — jf2c00170_si_001.pdf [file jf2c00170_si_001.pdf]
